# Better outcomes of robotic liver resection in segment VII/VIII compared to open and laparoscopic approach

**DOI:** 10.1007/s11701-026-03328-2

**Published:** 2026-04-07

**Authors:** Tiffany Joeng, Charing Ching Ning Chong, Eugene Yee Juen Lo, Hon Ting Lok, Janet Kung, John Wong, Kit Fai Lee, Kenneth Siu Ho Chok

**Affiliations:** Department of Surgery, Prince of Wales Hospital, Sha Tin, Hong Kong China

**Keywords:** Hepatocellular Carcinoma, Liver resection, Laparoscopic, Robotic, Superolateral segments

## Abstract

**Background:**

With the introduction of minimally invasive surgery, laparoscopic and robotic approaches have become increasingly popular for liver resections. However, few studies have compared the perioperative outcomes of open (OLR), laparoscopic (LLR), and robotic liver resections (RLR) together at once for lesions located in the superior parts of the right anterior and posterior segments, S7 and S8. This study reports data for S7 and S8 up to 5 years post-operation.

**Methods:**

Perioperative data of S7 and S8 resections performed at Prince of Wales Hospital from January 2014 to December 2024 were retrieved. For statistically significant variables (*p* < 0.05), pairwise comparisons using Bonferroni correction was performed. Kaplan-Meier method was used for survival analysis.

**Results:**

A total of 162 resections were retrospectively analysed, including 137 open, 14 laparoscopic, and 11 robotic liver resections. Robotic resections achieved the shortest post-operative length of stay (OLR: 7, LLR: 6, RLR: 4 days, *p* < 0.001) out of the 3 approaches and a 0.0% major complication rate (OLR: 11.7%, LLR: 0.0%, RLR: 0.0%, *p* = 0.385). Laparoscopic resections had the shortest operative duration (OLR: 175, LLR: 160.5, RLR: 190 min, *p* < 0.001) and least amount of intra-operative blood loss (OLR: 230, LLR, 60.5, RLR, 100 mL, *p* = 0.401).

**Conclusions:**

Despite an increased operating time, robotic liver resection demonstrated lower rates of major complications and shorter post-operative length of stay compared to open and laparoscopic resections. R1 resection rate and intra-operative blood loss were also comparable to laparoscopic liver resection. This solidifies robotic liver resection as a safe and effacious alternative to its open and laparoscopic counterparts.

## Introduction

Liver resection is currently the first-line curative treatment for eligible patients with hepatocellular carcinoma (HCC) and hepatic metastasis of colorectal cancer (CRC) [1, 2]. While open liver resection (OLR) remains the traditional approach, indications for laparoscopic surgery have expanded rapidly over the past few decades, and now laparoscopic liver resection (LLR) stands as an established alternative to OLR. LLR offers various benefits over OLR, including a smaller incision scar, better visualisation of the lesion and its surroundings, higher precision of the laparoscope, less blood loss and risk of infection, all of which have translated into improved perioperative outcomes and equivalent oncological outcomes to OLR [3–5]. With the advancement of modern technology, robotic surgery has been made more and more common, and robotic liver resection (RLR) is now an option in many tertiary centres [6]. Robotic surgery offers a solution to some of the problems encountered with laparoscopy, such as increased stability and range of motion of the robotic arm, 3D visualisation of the lesion, reducing the difficulty of intracorporal suturing and haemostasis. It also improves surgeon ergonomics. Meta-analyses have reported comparable perioperative and survival outcomes between LLR and RLR [7, 8].

A major challenge in laparoscopic liver resection is that of the superolateral liver, corresponding to S7 and 8 under the Couinaud classification. As they are covered by the costal cage and surrounded by the right and/or middle hepatic veins, exposure to S7 and 8 is poor and accessibility is limited, leading to high rates of intraoperative bleeding and complications. Compared to the anterolateral liver, laparoscopic resections of the superolateral liver have consistently resulted in longer operating times and higher rates of Pringle manoeuvre to control bleeding [9].

Currently, there is limited published data comparing the perioperative and survival outcomes of open, laparoscopic, and robotic liver resection together. 2 propensity score analyses found comparable 5-year overall survival (OS) rates across the 3 modalities; and reported RLR to have the longest operative duration but lowest rate of major complication and open conversion rate [10, 11]. Moreover, much of the current research focuses on the posterolateral liver (encompassing S1, 4a, 7 and 8) instead of S7 and 8 specifically [12–14]. The International consensus statement on robotic hepatectomy surgery in 2018 stated there was low level of evidence for the recommendation: ‘Robotic hepatectomy is as safe and feasible as traditional open hepatectomy’, reflecting that more studies are warranted on this topic [15].

The aim of this study was to compare the perioperative and survival outcomes of OLR, LRL, and RLR by evaluating data from a single-centre experience.

## Materials and methods

### Patient inclusion criteria

In this single-centre retrospective study, data on all patients who underwent S7 and 8 liver resections at our institution from January 2014 to December 2024 were collected. Only primary resections were included. Patients were included regardless of the indication for resection.

## Data collected

Data was retrieved from electronic health records, including baseline patient characteristics (age, sex, Hepatitis B or C status, Child’s grade, and cirrhosis status), haematological data from pre-operative blood, tumour (solitary tumour status and tumour size), intra-operative details (type of operation, rate of conversion to OLR, operative duration, operative blood loss, intra-op blood transfusion, and R1 resection rate), and post-operative outcomes (complications, post-op hospital stay, and in-hospital mortality). Post-operative complications were graded in accordance to the Clavien-Dindo classification, with complications above grade 2 considered major [16]. After discharge, patients were followed up at out-patient clinic, and 1-, 3-, 5-year OS rates were retrieved.

Primary outcomes included operative duration and operative blood loss. Secondary outcomes included both perioperative (rate of conversion to OLR, intra-op blood transfusion, R1 resection rate, post-op complications, post-op hospital stay) and oncological outcomes.

## Perioperative management and surgical procedures

Hepatic function assessment, including the Child Pugh Score and the indocyanine green (ICG) clearance test, was performed after the HCC was considered resectable as shown by ultrasonography, computed tomography, or magnetic resonance imaging.

Intraoperative ultrasonography was performed routinely to detect tumour invasion into the major branches of the portal or hepatic veins or any lesion in the contralateral lobe. This also allowed us to mark the line of parenchymal transection on the liver surface to obtain the optimal tumour-free margin and to avoid a major hepatic vein at the transection plane. Intraoperative ultrasonography was repeated during parenchymal transection to define the spatial relationship of the transection plane to the tumour so that the transection plane did not approach the tumour.

After surgery, the patients were monitored in the high dependency unit or intensive care unit with attention to fluid balance, oxygenation, and tissue perfusion. All patients were given broad-spectrum antibiotics for 7 days. Parenteral nutrition consisting of branched-chain amino acid-enriched solution, low-dose dextrose, medium- and long-chain triglycerides, phosphate, may be given in all patients with advanced cirrhosis and was continued for 3 to 7 days by means of a central line inserted aseptically. Oral diet was encouraged once bowel sounds returned and the parenteral nutrition would be stopped afterwards.

### Statistical analysis

Continuous variables were expressed as medians and range, and categorical variables were expressed as count and percentage. Chi-squared test or Fisher’s exact test was performed for comparison of categorical variables. Kruskal-Wallis test was performed for the comparison of continuous variables. For statistically significant variables (*p* < 0.05), pairwise comparisons using Bonferroni correction was performed. Kaplan-Meier method was used for survival analysis and survival curves were compared using the log-rank test. P-value less than 0.05 was taken as the level of statistical significance. Statistical analysis was performed using IBM SPSS Statistics (Version 27).

## Results

In total, 162 patients were identified who underwent resection of S7 and 8 of the liver. Of these, 137 (84.6%) underwent OLR, 14 (8.6%) underwent LLR, and 11 (6.8%) underwent RLR. The median age was 59-70.5 throughout the 3 cohorts, and 117 patients were male (72.2%). There was no significant difference in Child’s grade or ASA across cohorts, and overall 161 patients (99.4%) had Child’s grade A disease (*p* > 0.999). However, patients in the RLR cohort (54.5%) were more likely to have histologically confirmed cirrhosis than those undergoing OLR (44.5%) and LLR (42.9%) (*p* = 0.808). For pre-operative blood, there was no significant difference across the cohorts, except for albumin (*p* = 0.011). The pathological diagnosis was hepatocellular carcinoma (HCC) in 63.6–85.7% of patients across the cohorts, and colorectal metastases in 14.3–36.4% of patients across the cohorts. A majority of patients had a solitary tumour, and there was a significant difference in median tumour size, which was smaller in LLR (1.8 cm) and larger in OLR (2.5 cm) and RLR (2.5 cm) (*p* = 0.008). (Table 1)

**Table 1:** Patients’ demographics and tumour characteristics

	Open (*n* = 137)	Lap (*n* = 14)	Robotic (*n* = 11)	*P*-value
Age	65 (30–89)	70.5 (58–83)	59 (41–74)	0.008*
Sex (M)	98 (71.5%)	10 (71.4%)	9 (81.8%)	0.874
Hepatitis B or C	93 (67.9%)	12 (85.7%)	4 (36.4%)	0.357
Child’s grade				
A	136 (99.3%)	14 (100.0%)	11 (100.0%)	> 0.999
B	1 (0.7%)	0 (0.0%)	0 (0.0%)	> 0.999
ASA				
1	4 (2.9%)	0 (0.0%)	0 (0.0%)	> 0.999
2	107 (78.1%)	10 (71.4%)	8 (72.7%)	0.682
3	26 (19.0%)	4 (28.6%)	3 (27.3%)	0.467
Cirrhosis	61 (44.5%)	6 (42.9%)	6 (54.5%)	0.808
Pre-op Blood Hb Platelet WCC INR Albumin Bilirubin ALT Cr	13.6 (8.2–17.2) 175 (55–640) 5.8 (2.8–13.7) 1.00 (0.86–1.34) 38 (24–47) 10 (3–34) 26 (10–101) 79 (41–213)	13.6 (10.5–15.4) 196.5 (112–274) 6.5 (3.5–14) 0.99 (0.93–1.27) 36 (29–40) 9.5 (3–39) 34.5 (15–162) 73 (54–131)	13.7 (11.7–16.4) 171 (77–306) 6.0 (3.8–9.9) 1.00 (0.93–1.15) 37 (28–47) 8 (4–45) 27 (15–106) 79 (56–200)	0.844 0.532 0.498 0.943 0.011* 0.953 0.288 0.979
Pathological diagnosis HCC CR Met CA Corpus Met CA Pancreas Met CA Stomach Met CholangioCA Hemangioma Hepatic adenoma Granulomatous inflammation IgG4 disease No viable tumour	101 (73.7%) 23 (16.8%) 1 (0.7%) 1 (0.7%) 1 (0.7%) 4 (2.9%) 2 (1.5%) 1 (0.7%) 1 (0.7%) 1 (0.7%) 1 (0.7%)	12 (85.7%) 2 (14.3%) 0 (0.0%) 0 (0.0%) 0 (0.0%) 0 (0.0%) 0 (0.0%) 0 (0.0%) 0 (0.0%) 0 (0.0%) 0 (0.0%)	7 (63.6%) 4 (36.4%) 0 (0.0%) 0 (0.0%) 0 (0.0%) 0 (0.0%) 0 (0.0%) 0 (0.0%) 0 (0.0%) 0 (0.0%) 0 (0.0%)	0.459 0.266 > 0.999 > 0.999 > 0.999 > 0.999 > 0.999 > 0.999 > 0.999 > 0.999 > 0.999
Solitary tumour	123 (89.8%)	13 (92.9%)	9 (81.8%)	0.654
Tumour size (cm)	2.5 (0.5–8.0)	1.8 (1.0–2.8)	2.5 (1.5–7.9)	0.008*

There was little variation in the type of operation performed for the different cohorts (see Table 2). 100% of patients who underwent LLR and RLR had a wedge resection, compared to 93.4% in the OLR cohort (*p* > 0.999). The remaining 6.6% of OLR patients had a segmentectomy (*p* > 0.999). One patient in the LLR cohort (7.1%) and 1 patient in the RLR cohort (9.1%) had to convert to open resection (*p* > 0.999). There was a difference seen in the median operation duration, which was shortest for LLR (160.5 min), followed by OLR (175 min), and longest for RLR (190 min) but was not statistically significant ( p=0.401). Intra-operative blood loss was significantly highest in OLR (230 mL), followed by RLR (100 mL) and lowest in LLR (60 mL)(P<0.001). Differences in intra-operative blood transfusion were also not significant (*p* = 0.529). The R1 resection rate was 6.6% in OLR, 7.1% in LLR, and 27.3% in RLR with no significant variation (*p* = 0.071). (Table 2)

**Table 2:** Intra-operative data

	Open (*n* = 137)	Lap (*n* = 14)	Robotic (*n* = 11)	*P*-value
Type of operationSegmentectomyWedge resection	9 (6.6%) 128 (93.4%)	0 (0.0%) 14 (100.0%)	0 (0.0%) 11 (100.0%)	> 0.999 > 0.999
Convert open	NA	1 (7.1%)	1 (9.1%)	> 0.999
OT duration (min)	175 (70–345)	160.5 (100–275)	190 (116–358)	0.401
OT blood loss (ml)	230 (10–3800)	60.5 (20–600)	100 (20–400)	< 0.001*
Intra-op blood transfusion	7 (5.1%)	0 (0.0%)	1 (9.1%)	0.529
R1 resection	9 (6.6%)	1 (7.1%)	3 (27.3%)	0.071

**Fig. 1:**
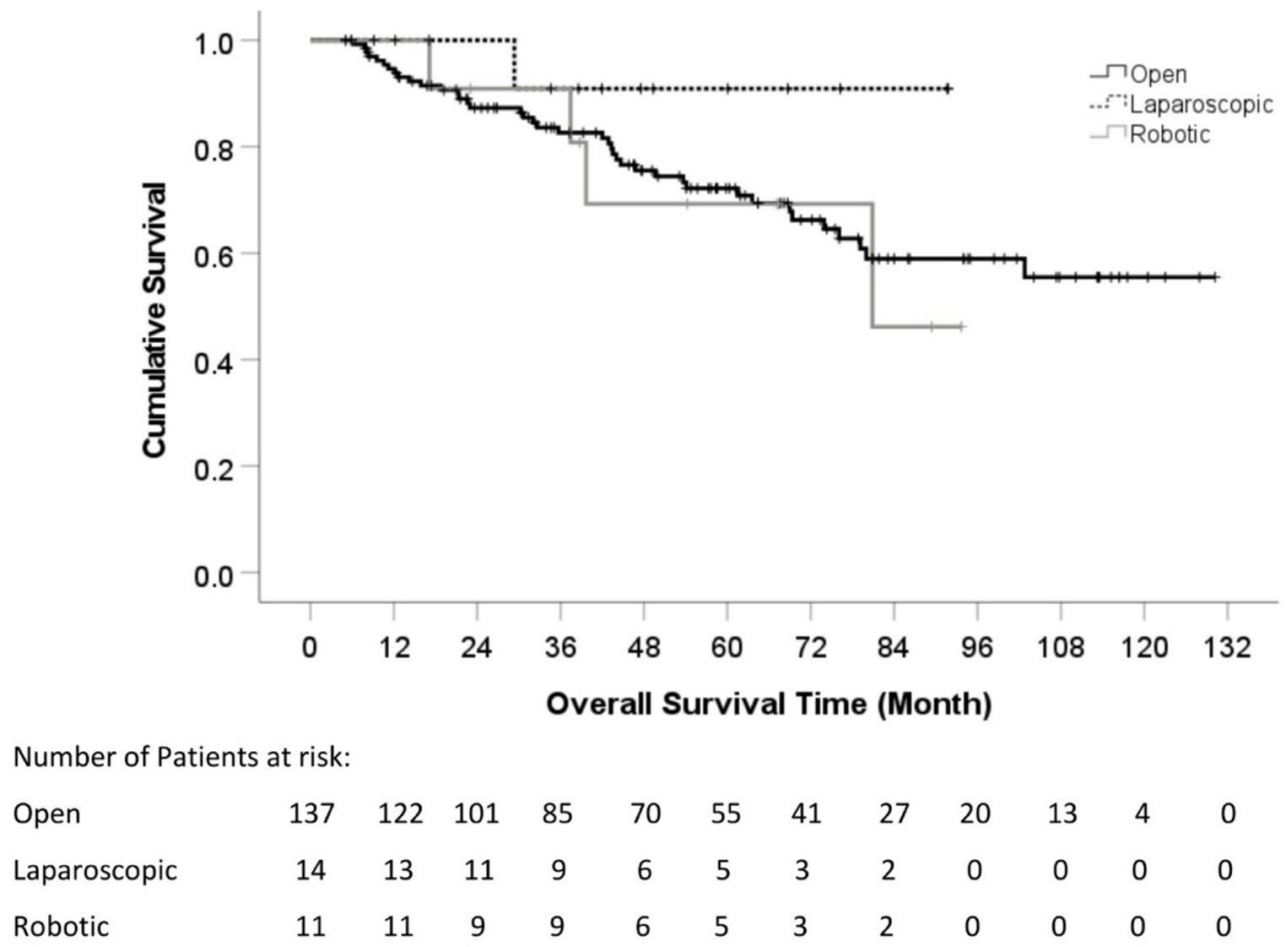
Overall Survival Log-rank test: P-value = 0.368

There was no significant difference in either total complications (*p* = 0.259) or major complications (Clavien-Dindo grade > 2) (*p* = 0.385) across cohorts (Table 3). However, there was a significant difference in post-operative hospital stay. The post-operative hospital stay of OLR, LLR and RLR were 7 days, 6 days, and 4 days, respectively (*p* < 0.001). (Table 4) Overall survival (OS) rate was 72.2%, 90.9% and 69.3% at 5 years for OLR, LLR and RLR respectively. (Fig. 1)

**Table 3:** Post-operative data

	Open (*n* = 137)	Lap (*n* = 14)	Robotic (*n* = 11)	*P*-value
Complication	40 (29.2%)	2 (14.3%)	1 (9.1%)	0.259
Types of complications : Pleural effusion Chest infection Ileus Ascites AROU Intra-abdominal collection Atelectasis Delirium Septicaemia Adrenal gland haemorrhage AF IO MI Pneumoperitoneum Renal failure TIA	14 (10.2%) 12 (8.8%) 8 (5.8%) 5 (3.6%) 4 (2.9%) 3 (2.2%) 2 (1.5%) 2 (1.5%) 2 (1.5%) 1 (0.7%) 1 (0.7%) 1 (0.7%) 1 (0.7%) 0 (0.0%) 1 (0.7%) 0 (0.0%)	0 (0.0%) 1 (7.1%) 0 (0.0%) 0 (0.0%) 1 (7.1%) 0 (0.0%) 0 (0.0%) 0 (0.0%) 0 (0.0%) 0 (0.0%) 0 (0.0%) 0 (0.0%) 0 (0.0%) 0 (0.0%) 0 (0.0%) 1 (7.1%)	0 (0.0%) 0 (0.0%) 0 (0.0%) 0 (0.0%) 0 (0.0%) 0 (0.0%) 0 (0.0%) 0 (0.0%) 0 (0.0%) 0 (0.0%) 0 (0.0%) 0 (0.0%) 0 (0.0%) 1 (9.1%) 0 (0.0%) 0 (0.0%)	0.415 0.843 > 0.999 > 0.999 0.572 > 0.999 > 0.999 > 0.999 > 0.999 > 0.999 > 0.999 > 0.999 > 0.999 0.068 > 0.999 > 0.999
Wound infection	1 (0.7%)	0 (0.0%)	0 (0.0%)	> 0.999
Major Complication (Clavien-Dindo grade > 2)	16 (11.7%)	0 (0.0%)	0 (0.0%)	0.385
Post-op hospital stays (day)	7 (3–32)	6 (3–10)	4 (3–8)	< 0.001*
In-hospital mortality	0 (0.0%)	0 (0.0%)	0 (0.0%)	> 0.999

**Table 4:** Pairwise comparisons of statistically significant variables (*p* < 0.05)

Variable	Pairwise comparison	*P*-value
Age	OLR vs. LLR	0.162
	OLR vs. RLR	0.067
	LLR vs. RLR	0.002*
Albumin	OLR vs. LLR	0.038*
	OLR vs. RLR	0.658
	LLR vs. RLR	0.165
Tumour size (cm)	OLR vs. LLR	0.007*
	OLR vs. RLR	> 0.999
	LLR vs. RLR	0.052
OT blood loss (ml)	OLR vs. LLR	0.002*
	OLR vs. RLR	0.038*
	LLR vs. RLR	> 0.999
Post-op hospital stays (day)	OLR vs. LLR	0.134
	OLR vs. RLR	< 0.001*
	LLR vs. RLR	0.376

## Discussion

Few studies have compared the perioperative benefits of RLR, LLR and OLR together for the superolateral liver (S7, 8) specifically [17]. In this study, RLR achieved lower rates of major complications and shorter post-operative length of stay compared to LLR and OLR, as well as comparable rates of open conversion to LLR, solidifying RLR as a safe and efficacious approach to complex resections of S7 and 8.

Complex resections, historically performed via OLR and associated with higher morbidity, are now increasingly undertaken laparoscopically, offering numerous perioperative benefits [3–5]. However, the efficacy of LLR remains a question for lesions located in challenging locations like superolateral segments. RLR offers surgeons enhanced precision and control as well as improved 3D visualisation, hence, it has the potential to overcome the constraints of LLR. The foremost question of this study is therefore whether robotic surgery can achieve comparable if not more favourable perioperative outcomes than laparotomy for superolateral resections, thereby paving the way to expand the indications of RLR.

In our study, we observed a shorter length of post-operative hospital stay after RLR compared to LLR and OLR, which was statistically significant (*p* < 0.001). Bonferroni correction was performed, after which a significant difference was noted between OLR and LLR. Existing literature has reported inconsistent findings on whether length of stay is longer in RLR or LLR [7, 10, 11, 18, 19]. However, a multivariate regression analysis performed by Xie et al. [20] showed that RLR was independently associated with lower hazard of prolonged LOS (HR = 0.592, 95% CI: 0.321–1.093). It should also be noted that in metanalyses comparing LLR and RLR for the superolateral segments, there was no statistically significant difference in the length of post-operative hospital stay [21, 22]. Rate of complications was lower in RLR than LLR and OLR, while there no major complications (Clavien-Dindo grade > 2) occurred in both RLR and LLR compared to a rate of 11.7% in OLR.

Low cost-effectiveness of RLR is often cited as a major obstacle in the broad implementation of robotic surgery. A study by Knitter et al. [23] analysed the costs of liver resection, and found that length of stay (HR [95% CI] = 8.8), development of major complications (HR [95% CI] = 2.9) length of stay (HR [95% CI] = 5.4) were all independently associated with higher costs. Despite having a longer operative time, this study demonstrated that RLR has significantly shorter length of stay than LLR and OLR, as well as a low rate of major complications. Although robotic approach may have higher intraoperative costs, the total costs for robotic hepatectomy is not higher than that of laparoscopic approach due to the reduction in postoperative complications and hospital stay durations, particularly for complex cases. While our study did not investigate cost-effectiveness, studies based in the United States and Singapore have compared OLR, LLR and RLR together, finding that RLR had the highest total and procedural costs but the lowest hospitalization costs; or the highest ‘cost-effectiveness’ when factoring in cost per readmission [18, 19]. Combined with the favourable perioperative and survival outcomes, this solidifies RLR as a feasible alternative to LLR and OLR.

Our results align with prior literature demonstrating that RLR is associated with longer operative time, higher intraoperative transfusion requirements, and a higher R1 resection rate when compared with LLR and OLR [10, 11]. Robotic surgery takes a significantly longer operative time due to the additional docking time of the robotic system and lack of a dedicated ultrasonic aspirator for parenchymal resection. Surgeons have to switch between instruments such as bipolar forceps for vascular and biliary dissection, and daVinci^®^ Harmonic ACE™ (Ethicon, Somerville, NJ, USA) for parenchymal transection which will lead to increased operation time [24]. Nevertheless, numerous studies have demonstrated that the cumulative operative duration of RLR decreases with surgeon experience [25–27]. An analysis of 100 consecutive RLR cases performed by a single surgeon showed that cumulative operating time decreased by the 31st case, and stabilised by the 66th case [28].

While the percentage of R1 resection seems much higher in RLR (17.3%) than LLR (7.1%), the actual number of cases with R1 resection does not differ much (3 in RLR, 1 in LLR), and the difference is not statistically significant (*p* = 0.071). We believe the difference in rate is due to the small sample size rather causing a large standard error of estimation which also accounts for the low statistical significance. One could alternatively hypothesise the higher R1 rate is due to relative surgeon inexperience in handling robotic surgery. However, all 4 R1 cases occurred in the early phase irrespective of technique.

Theoretically, the robotic surgery camera and use of indocyanine green fluoroscopy offers better visualisation of the lesion and its surroundings than that of the laparoscope, which could reduce injury to nearby blood vessels. This is supported by meta-analyses which found significantly less blood loss after RLR compared to LLR, though no significant differences in the use of Pringle’s manoeuvre and vascular clamp [8, 29]. Interestingly, our data showed that the median intraoperative blood loss and rate blood transfusion for RLR were lower than in OLR, but higher than in LLR, though the difference was not statistically significant. This may be attributed to the formation of pneumoperitoneum, which increases intra-abdominal pressure and venous pooling [30].

A point to note is the low open conversion rate of both LLR and RLR in this cohort, with only 1 case in each arm each having to convert to open hepatectomy – owing to the tumour location being inconvenient for laparoscopic resection (LLR) and equipment failure (RLR) respectively. This minimises the dilution of purported benefits of minimally invasive surgery over OLR. While the rates of open conversion of LLR and RLR were comparable in this study, other studies generally reported lower rates of open conversion for RLR than LLR. Data from O’Connell et al. [11] found that open conversion occurred in 21% of LLR and 0% of RLR cases, suggesting effect of a learning curve in surgeons who started performing LLR before proceeding to RLR. This is supported by a metanalysis from Liang et al. [22] focusing on the posterosuperior segments (S4a, 7, 8), which reported a 2.8% conversion rate in RLR and 7.0% in LLR. Intraoperative bleeding is a main reason for conversion in minimally invasive liver resection, and converted cases are associated with unfavourable rates of post-operative morbidity and mortality [31]. As described above, RLR can theoretically reduce bleeding rate, which translates to reduced open conversion rate.

This study has also several limitations. As a single-centre retrospective cohort study, it is subjected to potential selection bias due to its non-randomised protocol. There is a relatively low number of patients compared to other cohorts, especially in the LLR and RLR arms (14 and 11 patients respectively). The number of patients is also disproportionately skewed towards the OLR arm which included 137 patients. Patients were selected from a timespan of 10 years (2014–2024), where there may have been a learning curve of hepatobiliary surgeons; it may be possible that earlier results do not accurately reflect the current outcomes of LLR or RLR performed by the same surgeon. It also does not report on long-term survival data beyond 5 years due to limited follow-up data. Certainly, in the future, more trials should be performed with larger sample size for LLR and RLR, as well as with longer follow-up.

## Conclusion

Our study confirms that despite an increased operating time, RLR offers lower rates of major complications and shorter post-operative length of stay over LLR and OLR. There was no significant difference between the 5-year OS rates of the 3 approaches. Though the sample size is relatively small, these results nevertheless are encouraging and suggest RLR may be a safe alternative to LLR and OLR for resections of the superolateral liver segments. Further studies are warranted to confirm these results with a larger study population and investigate the long-term morbidity and mortality of these 3 approaches.

## Data Availability

Data generated is from our institutional database.
